# Biparametric MRI in prostate cancer during active surveillance: is it safe?

**DOI:** 10.1007/s00330-024-10770-z

**Published:** 2024-04-24

**Authors:** Iztok Caglic, Nikita Sushentsev, Tom Syer, Kang-Lung Lee, Tristan Barrett

**Affiliations:** 1 Department of Radiology, Cambridge University Hospitals NHS Foundation Trust, Addenbrooke’s Hospital, Cambridge, United Kingdom; 2 Department of Radiology, University of Cambridge, Cambridge, United Kingdom; 3 Department of Radiology, Taipei Veterans General Hospital, Taipei, Taiwan; 4 School of Medicine, National Yang Ming Chiao Tung University, Taipei, Taiwan

**Keywords:** Prostate cancer, MRI, Active surveillance, Biparametric

## Abstract

**Abstract:**

Active surveillance (AS) is the preferred option for patients presenting with low-intermediate-risk prostate cancer. MRI now plays a crucial role for baseline assessment and ongoing monitoring of AS. The Prostate Cancer Radiological Estimation of Change in Sequential Evaluation (PRECISE) recommendations aid radiological assessment of progression; however, current guidelines do not advise on MRI protocols nor on frequency. Biparametric (bp) imaging without contrast administration offers advantages such as reduced costs and increased throughput, with similar outcomes to multiparametric (mp) MRI shown in the biopsy naïve setting. In AS follow-up, the paradigm shifts from MRI lesion detection to assessment of progression, and patients have the further safety net of continuing clinical surveillance. As such, bpMRI may be appropriate in clinically stable patients on routine AS follow-up pathways; however, there is currently limited published evidence for this approach. It should be noted that mpMRI may be mandated in certain patients and potentially offers additional advantages, including improving image quality, new lesion detection, and staging accuracy. Recently developed AI solutions have enabled higher quality and faster scanning protocols, which may help mitigate against disadvantages of bpMRI. In this article, we explore the current role of MRI in AS and address the need for contrast-enhanced sequences.

**Clinical relevance statement:**

Active surveillance is the preferred plan for patients with lower-risk prostate cancer, and MRI plays a crucial role in patient selection and monitoring; however, current guidelines do not currently recommend how or when to perform MRI in follow-up.

**Key Points:**

- *Noncontrast biparametric MRI has reduced costs and increased throughput and may be appropriate for monitoring stable patients.*
- *Multiparametric MRI may be mandated in certain patients, and contrast potentially offers additional advantages.*
- *AI solutions enable higher quality, faster scanning protocols, and could mitigate the disadvantages of biparametric imaging.*

## Introduction

Active surveillance (AS) is a strategy recommended by the European Association of Urology (EAU) for patients presenting with low or favourable intermediate-risk prostate cancer (PCa) [1]. AS involves identifying optimal timings for curative treatments based on individual cancer grade, stage, and patient preference to safely delay or avoid radical treatments along with their potential side effects whilst maintaining oncological outcomes. The overall safety of AS in patients with clinically localised PCa has been established through several randomised controlled trials, such as ProtecT and PIVOT [2, 3], which relied on systematic biopsies as means of patient risk stratification. Disease-specific mortality in both trials remained very low despite the potential for understaging and undergrading in the pre-MRI era; hence, the argument for AS in low- and intermediate-risk disease is strong [4].

AS is implemented through regular monitoring of prostate-specific antigen (PSA), along with repeated MRI examinations and protocol-driven repeat biopsies. Eligibility criteria for AS vary across international, national, and local guidelines. These criteria are primarily based on factors such as Gleason score, cancer biopsy core volume, PSA levels, and clinical stage. Typically, AS is recommended for Gleason grades ≤ 3 + 3, PSA ≤ 10 ng/mL, and T-stage ≤ 2a, with some guidelines further supporting its use for favourable intermediate-risk Gleason 3 + 4 disease [1, 5, 6].

MRI is now considered a crucial baseline assessment for AS eligibility and for ongoing monitoring [7]. Baseline MRI aids in lesion detection and targeted biopsy, improving initial risk classification and reducing the likelihood of under-sampling compared to non-targeted biopsy approaches [8]. A negative MRI is more likely to indicate lower-grade pathology better suited for AS [9]. Conversely, the presence of a PI-RADS score of 4 or 5 lesion predicts higher-grade disease, which may render patients ineligible for AS [10]. Local staging with MRI to confirm organ-confined disease is also important for AS eligibility. Meta-analyses of MRI in AS follow-up report a high pooled NPV ranging from 0.81 to 0.88, and lower PPV ranging from 0.37 to 0.50, which may be due to several factors, including variability in measurements, subjective assessment of lesion conspicuity and lack of quantitative thresholds [11, 12]. The relatively low PPV for identifying disease progression poses a risk of over-treatment, thus MRI progression alone should not be the only trigger for repeat biopsy or even treatment. Instead, it should be considered among other clinical factors in a decision-making process, such as dynamic changes of PSA and PSA density [7, 13]. In this article we explore the current role of MRI in AS and how this should be performed, in particular addressing the need for contrast-enhanced sequences.

## Mutiparametric versus Biparametric MRI

Currently, PI-RADS v2.1 guidelines recommend a full multiparametric MRI (mpMRI) protocol, including T_2_-weighted (T2W), diffusion-weighted imaging (DWI), and dynamic contrast-enhanced (DCE) sequences for patients presenting with suspected localised or locally advanced prostate cancer [14]. The risks linked to Gadolinium-based contrast agents usage include allergic reactions, with severe adverse reactions occurring in only 0.005% [15]. Gadolinium has also been shown to deposit in a dose-dependent manner within the globus pallidus and the dentate nucleus and has been implicated in causing nephrogenic systemic fibrosis (NSF) in individuals with renal failure [16–20]. Biparametric MRI (bpMRI) comprising T_2_- and diffusion-weighted sequence without contrast administration presents several potential advantages to mpMRI, including cost savings, greater flexibility in scanning patients out-of-hours, and eliminating potential for side effects of contrast agents [21–24].

In the initial diagnostic setting, a biparametric approach may be preferred if high-quality imaging and expert interpretation are available, alongside the potential for patient recall or on-table monitoring [23, 25]. In 2018, 34.9% of centres in the UK performed bpMRI as a default protocol in biopsy-naïve patients [26]. This practice is supported by a systematic review of 6055 patients in 44 studies showing equivalent performance for bpMRI and mpMRI, with a slight increase in sensitivity of 0.87 versus 0.84 for mpMRI offset by a reduced specificity of 0.72 compared to 0.75 for bpMRI [27]. However, it should be noted that the included studies were exclusively retrospective or single-centre prospective studies, with heterogeneous inclusion criteria, and where mpMRI ultimately dictated the biopsy decision [28]. Furthermore, a randomised controlled trial of 311 biopsy-naïve patients showed a 9.2% higher detection of clinically significant PCa (csPCa) for mpMRI, although this did not reach significance, the study may have been underpowered [29]. Prospective head-to-head studies are currently recruiting to further address this issue [30, 31].

## The PRECISE role of MRI in AS

During the monitoring phase of AS, EAU guidelines recommend the use of MRI in men with rising PSA [1]. However, the frequency and intensity of MRI follow-up are not defined yet. This may be performed at routine, pre-determined time points [32], or may be triggered by PSA kinetics or clinical signs of progression. In addition, some centres have advocated their own institutional risk-based tailored approach, which could inform more personalised AS strategies [33, 34]. The Prostate Cancer Radiological Estimation of Change in Sequential Evaluation (PRECISE) recommendations provide a standardised tool and scoring system for assessing the likelihood of radiological progression on MRI during AS [35]. The PRECISE system assigns 5 categories of stability or change in MRI, with score 3 representing radiological stability, scores 1–2 reduction of previously suspicious MRI features, and PRECISE 4–5 progressive disease, which typically triggers a repeat biopsy (Table 1). Imaging signs of progression include increased conspicuity, increase in PI-RADS score, appearance of new lesions, increased lesion size, or worsening disease stage. Notably, the role of DCE is not explicitly discussed, unlike with T2W and DWI which are the dominant sequences and from which lesion size, a key metric, is obtained. However, the parameter of conspicuity inherently relates also to DCE and should be evaluated in relation to the background of the gland. This becomes especially apparent in patients with prostatitis, as the diffuse changes observed on mpMRI (particularly on T2W and DCE) can pose challenges in delineating the exact extent of the lesion. However, the recommendations acknowledge the absence of robust data on which to base a threshold for a significant change in size or conspicuity. A recent single study by Sushentsev et al recommended a 20% increase in the size on T2W and/or a 10% decrease in apparent diffusion coefficient (ADC) values; however, the study did further focus on DCE. Despite certain limitations of the PRECISE assessment system [36], a systematic review has shown a trend towards improved performance over institute-specific systems, likely due to the more objective categorisation of potential progression [11].

**Table 1:** PRECISE guidelines MRI assessment of radiological progression in AS follow-up

PRECISE category	Radiological features
**1**	Resolution of previous features suspicious on MRI
**2**	Reduction in volume and/or conspicuity of previous features suspicious on MRI
**3**	Stable MRI appearance: no new focal/diffuse lesions
**4**	Significant increase in size and/or conspicuity of features suspicious for prostate cancer
**5**	Definitive radiologic stage progression

Adapted from reference [35]

## Biparametric MRI in AS

The PRECISE guidelines were formed through a multidisciplinary consensus approach, and state that MRI protocols should meet the minimum criteria set by PI-RADS, but do not explicitly outline whether MRI should be performed with or without contrast administration. Sushentsev et al reported a 9.6-fold increase in the number of AS scans performed between 2010 and 2018 [37], consistent with trends in other tertiary referral centres in the US and Europe [38, 39], making shortened-protocols attractive for adequately managing the ever-growing demand on imaging services. Abbreviated protocols, which limit sequences to the axial plane can further reduce scan time and aid patient throughput [34]; Table 2. Such approaches have the added advantages of lower costs, reduced risk of gadolinium deposition and/or contrast reactions and may be better tolerated by patients.

The role of MRI in AS follow-up is substantially different from the initial diagnostic MRI. Patients have already been characterised with MRI, have a biopsy-proven diagnosis of lower-risk disease, and the paradigm is to assess for “radiologically significant” progression, defined as a PRECISE score 4–5 [35]. Aside from further MR imaging, patients will also have the clinical safety net of continuing outpatient clinic appointments, with regular PSA checks and protocol-driven repeat biopsies. Indeed, a recent PI-RADS committee narrative review suggested bpMRI as an option in patients undergoing routine AS follow-up [23]. However, contrast is mandated for patients at higher risk of progression due to fast PSA doubling times or changing clinical or pathologic status, wherein the balance between under-diagnosis and over-diagnosis leans toward the clinical priority of not missing significant cancer [23]. It would also be reasonable to perform mpMRI following interventions that alter the background signal or morphology of the gland, including transurethral resection of the prostate (TURP), holmium laser enucleation of the prostate (HoLEP), embolisation, finasteride, or radiotherapy for other pelvic malignancy (Table 3).

To date, there have been few studies assessing the added value of contrast-enhanced MRI over a biparametric approach in patients on AS; however, a prospective trial is currently recruiting [40]. Kortenbach et al showed that pre-biopsy bpMRI improved AS selection as compared to systematic TRUS biopsy [41], with the same group also demonstrating that bpMRI was non-inferior to mpMRI for AS enrolment based on early repeat imaging and biopsy [42]. Of the six studies reporting outcomes using the PRECISE scoring system, only one did not routinely use contrast for follow-up imaging [43], and none of the studies reported the individual MRI components contributing to the overall PRECISE score, thereby limiting the evaluation of the specific role of DCE during follow-up [11].

Two recent meta-analyses on MRI and PRECISE scoring during AS have been conducted, with 1 out of 15 and 0 out of 7 studies using bpMRI, respectively [11, 12]. The single study reporting on a bpMRI approach used mpMRI at baseline and an abbreviated 20-minute bpMRI protocol in follow-up. Despite this approach, the results are encouraging, with a higher NPV (0.96) than the pooled NPV (0.81–0.88), even with a long median follow-up of 52 months. Additionally, the study included patients with GG2 cancers, unlike the majority of other studies that included only men with GG1 disease. The PPV was also higher at 0.52, compared to the overall pooled PPV of 0.37–0.50 [40]. It is important to note that the study was conducted in a tertiary centre with expert prostate reporters and may not be transferrable to other settings. This caution aligns with findings from pre-biopsy MRI studies, indicating significantly worse diagnostic performance and confidence for less experienced readers when using bpMRI for lesion detection compared to mpMRI [44–46].

**Fig. 1:**
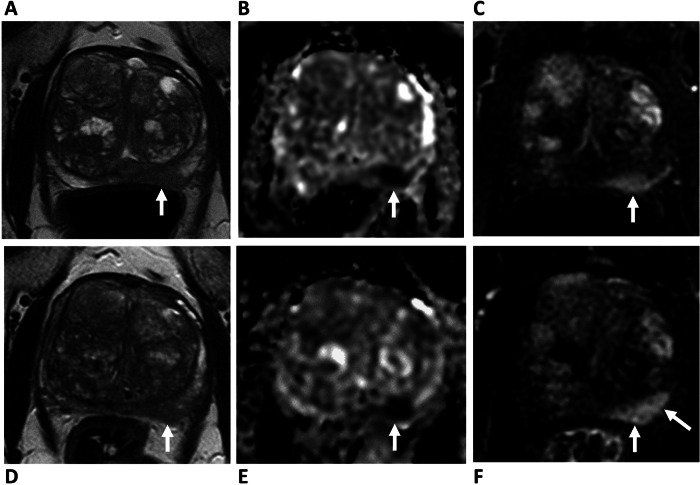
False positive DCE findings on AS follow-up. 74-year-old patient, presenting PSA 10.95 ng/mL. **A**–**C** Baseline MRI shows ill-defined T2 change (**A**) at the left mid-PZ, with a 12 × 9 mm area of marked restricted diffusion (**B**), with associated focal early enhancement (**C**), consistent with a PI-RADS 4 lesion (arrows). Targeted biopsy shows Gleason 3 + 4 = 7 (10% Gleason 4) in 3/3 cores. **D**–**F** MRI at 12 months, PSA 10.84 ng/mL. Stable conspicuity and size of lesion on T2 (**D**) and ADC maps (**E**), but increase in the degree of enhancement to 22 × 12 mm (**F**, arrows). PRECISE score 3—findings considered a false positive. The patient remains on AS

The NPV should be maximised in AS patients when the risk of treatment still overall outweighs the benefit of oncological control, and the prospect of repeated biopsies is the main reason for discontinuing AS by patients [7, 47–49]. The similar NPV of bpMRI compared to mpMRI suggests their equivalent potential for safely reducing the number of unnecessary biopsies and increasing patient adherence to AS protocols. The higher specificity of bpMRI compared to mpMRI for the detection of csPCa [27] may therefore be beneficial [11] and draws parallels with MRI-based prostate cancer screening, where a biparametric approach is advocated to help maximise specificity over sensitivity for the detection of significant cancer [50]. Notably, one of the main limitations of DCE is its relative non-specificity. In addition to tumours, both prostatitis and highly vascularised benign prostatic hyperplasia (BPH) nodules can result in rapid enhancement and wash-out, and false-positive results can also arise secondary to peri-lesional inflammatory change (Fig. 1). It is therefore essential to interpret DCE MRI results in combination with T2W and DWI sequences.

The current PRECISE system does not specifically define the appearance of new lesions [44–46]. Available studies typically categorised these as PRECISE 4 [36]. Notably, the PPV of MRI for predicting progression for new lesions was observed to be significantly lower at 24% compared to PRECISE scores 4–5 for pre-existing lesions (63%) in a recent study including 295 men [43]. Similarly, a study by Ghavimi et al showed that the majority of new lesions on follow-up scans were not of clinical significance and did not alter patient management [51]. A risk-tailored approach using bpMRI may therefore decrease the proportion of new PI-RADS 4 lesions, sparing men with low PSA-D from serial unnecessary biopsies [13].

**Fig. 2:**
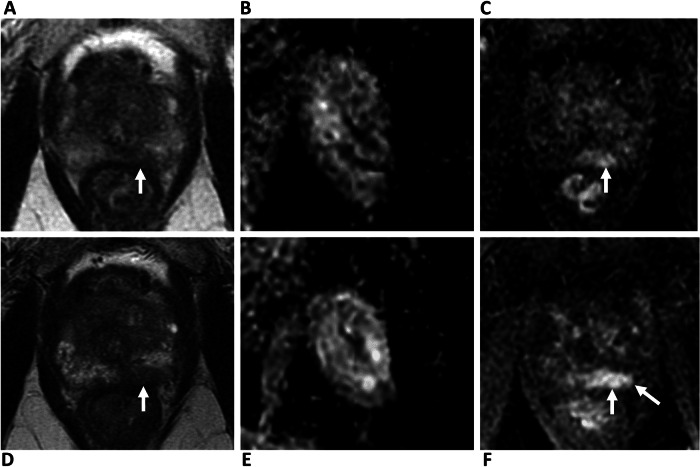
Value of DCE in patients with poor quality DWI. 71-year-old patient with a left THR, presenting PSA 6.01 ng/mL. **A–C** Baseline MRI: 14 × 5 mm PI-RADS 4 lesion in the medial left apex PZ with focal low T2 signal (**A**), nondiagnostic DWI due to THR (**B**), and focal early enhancement on DCE (**C**). Targeted biopsy shows Gleason score 3 + 4 = 7 (approximately 5% pattern 4), in 2/2 cores, 5 mm maximum tumour length. **D**–**F** MRI at 36 months with PSA 8.63 ng/mL. Increase in conspicuity on T2 (**D**), DWI remains nondiagnostic (**E**), but with a clear increase in the degree of enhancement on DCE to 21 × 7 mm (**F**, arrows). PRECISE score 4. Repeat biopsy shows Gleason score 3 + 4 = 7 (40% pattern 4), in 2/2 cores, 8 mm max tumour length. The patient treated with external beam radiotherapy

**Fig. 3:**
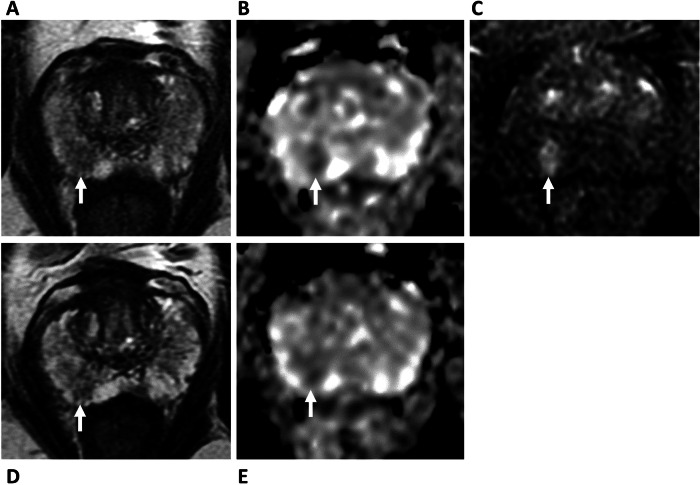
bpMRI versus mpMRI affecting PI-RADS score. 66-year-old patient, presenting PSA 5.88 ng/mL. **A**–**C** Baseline MRI shows ill-defined PI-RADS 3 change on T2 (**A**), with mild restricted diffusion on ADC (**B**) and *b*-value imaging (not shown), PI-RADS 3, with associated marked focal early enhancement, DCE positive (**C**, arrow). Overall PI-RADS score 3 + 1 = 4; targeted biopsy shows Gleason 3 + 4 = 7 (Pattern 4 = < 5%) extending for a maximum length of 4.2 mm. **D**, **E** MRI at 12 months, PSA 5.43 ng/mL. Stable appearances on Ts (**D**) and ADC maps (**E**, arrow); however, PI-RADS score 3, reduced from score 4 due to employment of a bpMRI only

There are also potential disadvantages of a biparametric approach (Table 4). DCE can act as a “safety net” sequence for image quality [52], and in the context of poor-quality imaging, PRECISE scoring cannot adequately be applied [36]; Fig. 2. DCE may also aid new lesion detection, particularly for lesion experienced readers [53], and may have advantages for accurate staging of the gland, particularly seminal vesicle invasion [54]. As expected, given the dominant and secondary sequence paradigm of PI-RADS, bpMRI will increase the number of indeterminate score 3 lesions in the PZ by 6.9–8.9% [46, 55, 56]. A baseline PI-RADS 4 lesion, up-scored by DCE (i.e. “3 + 1”) will be categorised as PI-RADS 3 on bpMRI follow-up assuming no change (Fig. 3). Theoretically, this change in PI-RADS score would be assigned as PRECISE category 2 due to a “reduction in the conspicuity of previous features suspicious on MRI”, despite the stability of the lesion itself. In practice, readers are likely to compare the biparametric alone sequences and assign a PRECISE score 3; again, such cases may help improve PPV by reducing the proportion of lesions scoring PI-RADS 4. Another consideration is the unknown effect on inter-reader agreement for PRECISE scoring at bpMRI, which to date has only been assessed using mpMRI, with Giganti et al reporting excellent agreement between two expert readers using mpMRI during AS [57].

**Table 2:** Local multiparametric, biparametric and abbreviated MRI protocols

Protocol	Localisers	Axial T1 FSE	Axial T2 FSE	Sagittal T2 FSE	Axial DWI large FOV	Axial DWI small FOV	DCE	Total time
Multiparametric	00:35	02:32	05:22	03:13	02:42	04:52	06:22	25:59
Biparametric	00:35	02:32	05:22	03:13	02:42	04:52	X	19:37
Abbreviated (AS)	00:56	02:32	05:22	X	02:42	X	X	11:32

*FOV* field-of-view, *FSE* fast spine echo

**Table 3:** Scenarios where an mpMRI approach is favoured in patients on AS

**Scenarios where mpMRI approach is recommended**
Patients at higher risk of progression (e.g. fast PSA doubling time, changing clinical or pathological status)
Pelvic metalwork (e.g. THR, surgical clips)
Prior prostate interventions (e.g. TURP, HoLEP, embolisation, finasteride, pelvic radiotherapy)
Patient-related factors affecting image quality (e.g. motion, rectal distension, high BMI)

*BMI* body mass index, *THR* total hip replacement

**Table 4:** Advantages and disadvantages of bpMRI in patients on AS

	Advantages	Disadvantages
**General**	• Avoids adverse Gadolinium reactions • Perform outside core hours • Reduced need for medical back-up • Cost savings	• Increase in number of nondiagnostic studies • Higher indeterminate PI-RADS 3 calls rate • Impact on reading and confidence of less experienced radiologists • DCE may improve T-staging accuracy
**During Follow-up**	• Higher specificity for csPCa may improve PPV of radiological progression • Abbreviated examination times and no cannula placement better tolerated by patients for multiple repeat studies • Increased capacity and patient throughput • Avoidance of false positive inflammatory change and downgrading lesions to PI-RADS 3 may improve PPV of radiological progression	• Direct comparison not possible for baseline DCE positive PZ lesions (PI-RADS score “3 + 1”) • No “safety net” in cases with reduced imaged quality may mean PRECISE scoring cannot be applied • DCE may aid new lesion detection • Unknown effect of omitting contrast on inter-reader agreement for PRECISE scoring

## Future directions

**Fig. 4:**
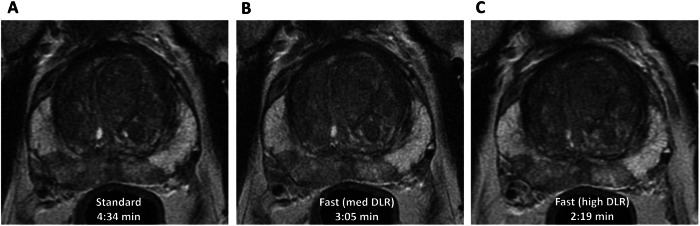
Faster acquisition by applying DLR. **A** Standard-of-care axial fast-recovery fast-spin-echo T2WI sequence, acquisition time 4:34 minutes. **B** **C** T2 acquisition with a reduced number of and retrospectively reconstructed with medium DLR (**B**) and high DLR (**C**), with resultant reduction in scan times to 3:05 minutes and 2:19 minutes, respectively, without compromising image quality

Artificial intelligence (AI) techniques are being routinely used in clinical settings as post-processing tools to reduce image noise using deep learning-based reconstruction (DLR) algorithms [58]. This offers the ability to further reduce scan time and/or to mitigate against the effects of poor image quality if a biparametric MRI approach is used (Fig. 4). The current version of Prostate Imaging Quality (PI-QUAL) does not allow for evaluating bp-MR image quality and PRECISE does not discuss the assessment of an AS follow-up MRI that is below acceptable quality thresholds [35, 59, 60]—both issues are likely to be addressed in subsequent versions of the guidelines scheduled for release in 2024 [61].

Recent studies pioneering the development of AI-assisted solutions for improving the baseline prediction and follow-up assessment of the risk of tumour progression on AS have to date, all used bpMRI approaches [62–65]. Importantly, two of these studies have shown comparable performance of fully quantitative AI models to expert-derived subjective PRECISE assessment, which has the potential for levelling up the performance of less experienced readers [63, 64]. Moreover, the combined predictive model using longitudinal bpMRI data together with serial PSA density has shown considerably higher performance compared to that of serial MRI results pooled across various centres [64, 66]. Furthermore, AI-assisted bpMRI assessment tools have the potential to provide automated readouts of measurable tumour characteristics, such as maximum diameter or volume [67, 68], which moving forward could help to make PRECISE-based image interpretation more objective [36].

## Summary

MRI has become an established tool for monitoring disease in patients on AS. Increased demand on imaging services makes the use of shortened MRI protocols attractive to aid patient throughput. Biparametric MRI enables this and has the added benefits of avoiding Gadolinium-associated side effects and is typically better tolerated by patients.

Data from studies involving biopsy-naïve patients suggests equivalent performance between bpMRI and mpMRI; however, the results of prospective studies in this cohort, including the PRIME and PACIFIC trials, are still awaited. The approach of bpMRI in the context of AS follow-up is arguably lower risk with the paradigm-shifting from that of lesion detection to the assessment of progression, and this is further supplemented by the safety net of ongoing clinical assessment. Indeed, a PI-RADS committee narrative review implies that bpMRI is a safe option in stable patients undergoing routine AS follow-up. The improved specificity afforded by bpMRI approaches could theoretically improve the PPV of MRI and safely reduce the need for repeat biopsies, where overall disease-specific mortality is known to remain very low. However, the use of contrast can afford advantages such as greater image quality, increased staging accuracy, and improved detection of new lesions.

In conclusion, retrospective single-centre studies and data derived from biopsy-naïve patient populations implies bpMRI to be a reasonable approach in AS follow-up, provided quality-control measures are met. The technique offers several benefits, and the recent development of AI solutions may help mitigate any potential disadvantages. However, further prospective data is required to fully establish the safety and efficacy of a bpMRI approach.

## Abbreviations

AS: Active surveillance
bp: Biparametric
mp: Multiparametric
PRECISE: Prostate Cancer Radiological Estimation of Change in Sequential Evaluation
PSA: Prostate-specific antigen
